# The Acceptability of Psychedelic‐Assisted Therapy Amongst Mental Health Consumers: Utilising the Theory of Planned Behaviour

**DOI:** 10.1111/inm.70010

**Published:** 2025-02-14

**Authors:** Eva Louie, Ellen Towers, Alyssa R. Morse, Joshua Watt, Zachary Bryant, Paul Haber, Kirsten Morley

**Affiliations:** ^1^ Discipline of Addiction Medicine, Central Clinical School, Faculty of Medicine and Health University of Sydney Sydney New South Wales Australia; ^2^ Edith Collins Centre (Translational Centre in Alcohol, Drugs & Toxicology) Sydney Local Health District & Royal Prince Alfred Hospital Camperdown New South Wales Australia; ^3^ Centre for Mental Health Research National Centre for Epidemiology and Population Health, The Australian National University Canberra Australian Capital Territory Australia; ^4^ The Matilda Centre for Research in Mental Health and Substance Use, Faculty of Medicine and Health University of Sydney Sydney New South Wales Australia

**Keywords:** acceptability, mental health consumer, psychedelic‐assisted therapy

## Abstract

Australian government approval has been granted for 3,4‐methylenedioxy‐methamphetamine (MDMA) treatment of post‐traumatic stress disorder and psilocybin for treatment‐resistant depression, but the process of translating psychedelic‐assisted therapies (PAT) into more widespread use is complex. Along with establishing the efficacy and feasibility of PATs, their acceptability amongst consumers is a crucial factor of successful implementation. This study utilised the Theory of Planned Behaviour to evaluate the acceptability of PATs amongst mental health consumers, identifying potential influences of these attitudes and predictors of PAT uptake. Participants completed an online survey between February and July 2023. Survey items evaluated consumer characteristics, acceptability of PAT (effectiveness, efficacy and social norms) and behavioural intentions to undertake PAT. The 254 participants had a mean age of 42.5 years (SD = 12.8) and 79.1% were female. Three quarters expressed a desire to access PAT. Acceptability scores indicated strong agreement regarding the effectiveness of PAT, social norms that moderately endorsed PAT and mixed feelings about its expected efficacy. Whilst univariate analyses indicated that previous psychedelic experience was associated with increased acceptability of PAT (*d*s = 0.63–0.80), multivariate analyses revealed that intentions to access PAT were associated with higher acceptability scores (*d*s = 0.37–1.32) and poorer experiences of conventional therapy (*d* = −0.31). Although a relatively large portion of participants had used psychedelics recreationally, the desire to access PATs was more strongly related to its acceptability, along with more negative experiences of conventional therapy. This implies that mental health consumers who are looking for alternatives to conventional therapy may view PATs as a desirable option, despite some safety reservations.

## 1 Introduction

Research interest in psychedelics has increased so rapidly that it is now considered one of the fastest growing areas of medical research (Petranker et al. 2020). Renewed clinical and academic interest in the potential benefits of psychedelic‐assisted therapy (PAT) is known as the ‘psychedelic renaissance’ (Godlee and Hurley 2016; Hari 2015). This has ushered in a new body of peer‐reviewed research, which addresses some of the methodological limitations of studies conducted prior to 1970 (e.g., absence of controls, unvalidated outcome measures, lack of statistical analyses, adverse outcomes not reported and lack of power; Rucker et al. 2018). Emerging evidence suggests that PATs may be effective for the treatment of depression (Carhart‐Harris et al. 2021; Davis et al. 2020), substance use disorders (Bogenschutz et al. 2015; Johnson et al. 2014), post‐traumatic stress disorder (Mitchell et al. 2021) and anxiety for patients facing terminal cancer (Griffiths et al. 2016; Grob et al. 2011; Ross et al. 2016). There is also some evidence that psychedelics may be effective for treating other conditions including eating disorders, migraine and cluster headaches, and early dementia (Siegel et al. 2021). One consistent finding across this research is that supportive counselling is an essential mediating factor when compared to using psychedelics as a standalone pharmacotherapy (Garcia‐Romeu and Richards 2018; Rucker et al. 2018).

This research interest is mirrored by that of the community and media to an extent that has scarcely been seen for any psychopharmacology or psychotherapy. Given the rising rates of mental illness, and the reported effectiveness of drugs that have been prohibited since the 1970s, there has been a growing interest in PATs within psychology and psychiatry (Davis et al. 2022; Barnett et al. 2018, 2023), and advocacy groups have been lobbying for the prompt translation of this evidence into community clinics (Williams et al. 2021). Government support has even been granted in Australia, with the Therapeutic Goods Administration approval for use of 3,4‐methylenedioxy‐methamphetamine (MDMA) for treating post‐traumatic stress disorder and psilocybin for treatment‐resistant depression (Therapeutic Goods Administration 2023). These approvals appear in the absence of any registered therapeutic product (https://www.tga.gov.au/resources/artg searched on 6 June 2024) and under complex regulations (https://www.tga.gov.au/products/unapproved‐therapeutic‐goods/mdma‐and‐psilocybin/supply‐manufacture‐or‐import‐mdma‐or‐psilocybin‐sponsors‐and‐manufacturers). Implementation of PATs should be done cautiously however, since factors ranging from training and accreditation to regulation and economics must be addressed in order to avoid problems with efficacy, safety and equity of access (given the extremely high costs of treatment), each of which can adversely affect the sustainability of PATs and experience of consumers (Williams et al. 2021). Experience gathered from efforts to implement PATs in research contexts has highlighted these complexities (Barnett et al. 2024).

Guidelines on the best practice methods for designing and evaluating complex interventions recommend an assessment of the feasibility and acceptability of the intervention before proceeding to subsequent stages of implementation (Skivington et al. 2021), since successful completion of this ‘pre‐implementation’ phase has been found to significantly contribute to implementation outcomes (Brown et al. 2014; Nadeem et al. 2018; Saldana et al. 2012, 2018; Alley et al. 2023). While most pre‐implementation investigations focus on the influence of ‘top down’ (national guidelines, governance and medical education) or ‘bottom up’ (provider and team) processes, the uptake of PAT is also likely to be influenced by perceived features of the intervention itself. These perceptions and views have been termed the ‘implementability’ of an intervention and can encompass the perspectives of relevant stakeholders including consumers (Klaic et al. 2022). When considering the implementability of an intervention, consumer‐level variables are of particular interest because they inevitably impact the outcomes of implementation efforts as active agents and consumers of healthcare (Chaudoir et al. 2013). As the intended beneficiaries of such interventions, there is also an imperative to respond to the attitudes and needs of the consumers who play an essential role in implementation (Pedersen et al. 2021).

One difficulty with evaluating the consumer‐level factors related to the implementability of any intervention is the lack of standardised or validated intervention acceptability questionnaires (Sekhon et al. 2017). The theoretical framework of acceptability (TFA) was devised in light of these disparities and includes seven generic constructs (affective attitude, burden, ethicality, intervention coherence, opportunity costs, perceived effectiveness and self‐efficacy; Sekhon et al. 2022). There are also some robust theories that have been used to predict evidence‐based practice adoption such as the widely used Theory of Planned Behaviour (TPB; Ajzen 1991; Presseau et al. 2011; Kortteisto et al. 2010; Aarons et al. 2009; Godin et al. 2008), which proposes that attitudes (favourable or unfavourable opinions about the target behaviour), perceived behavioural control (belief that one can perform the behaviour) and subjective norms (pressures to perform the behaviour) influence intentions to perform the behaviour (Ajzen 1991). The TPB has often been used to predict the behaviour of healthcare providers, and is commonly used to identify help‐seeking intentions (Tomczy et al. 2020).

To date, research published on the acceptability of PAT amongst mental health consumers is limited and has focused on attitudes and beliefs. For instance, findings from an Irish survey of mental health service user (*n* = 99) attitudes about the therapeutic potential of psychedelics and psilocybin therapy revealed a strong display of support for further research into the therapeutic potential of psilocybin (72%), a moderate agreement that it should be an approved medical treatment (59%) and moderate agreement that it would be accepted if a doctor recommended it (54%). Interestingly, participants who had a previous psychedelic experience had more favourable opinions towards psilocybin therapy (Corrigan et al. 2021). An Australian survey of attitudes towards PAT (*n* = 502) compared participants with and without self‐reported mental illness. Results indicated that participants with mental illness were more in favour of legalisation and therapeutic use of psychedelics and that knowledge and experience of psychedelics predicted positive attitudes towards legalisation and a willingness to engage with psychedelics (Nadeem et al. 2024). However, the focus of these studies was limited to knowledge and attitudes regarding PATs, and the absence of an implementation framework precluded measurement of a range of potential influencing factors.

Accordingly, there is a need for research into the acceptability of PAT amongst mental health consumers that is informed by implementation research. This study seeks to expand existing research by drawing upon the TPB to enhance the scope of measurement from a simple focus on attitudes and beliefs by: (1) describing the acceptability of PAT amongst mental health consumers (attitudes, social influences, expectancy and behavioural intentions), (2) identifying demographic characteristics and personal experiences (mental health, substance use, psychedelic use and conventional mental health treatment) that might influence mental health consumers' perceptions of the acceptability of PAT and (3) exploring potential associations of behavioural intentions to try PAT.

## 2 Methods

### 2.1 Ethical Approval

Ethics approval was obtained from the Sydney Local Health District Royal Prince Alfred Hospital (SLHD‐RPA) ethics committee. Protocol No: X22‐0243 and 2022/ETH01223.

### 2.2 Survey Design

Consumer characteristics including age, sex, postcode, mental health and substance use diagnoses, treatment history, previous experience with recreational use of psychedelics and any previous PAT were recorded. We defined psychedelics as follows: psilocybin, lysergic acid diethylamide (LSD), mescaline or 4‐Bromo‐, 2, 5‐dimethoxyphenethylamine (2C‐B), phencyclidine (PCP), Ketamine, Salvia, N, N‐Dimethyltriptamine (DMT) and 3,4‐methylenedioxy‐methamphetamine (MDMA). Information about participants' motives for using psychedelics was captured (using questions adapted from Lea et al. 2020), along with their subjective experience of previous treatment, psychedelics and PAT.

The TPB informed the questions related to acceptability of PAT, whereby knowledge and attitudes about the effectiveness of PATs (using questions sourced from Wildberger et al. 2017), and subjective norms (‘My peers support the use of recreational psychedelics’, ‘My peers support the use of psychedelics as a therapeutic tool’ and ‘People I respect endorse psychedelic‐assisted therapies’) were measured. Additionally, expectations about the efficacy of PAT were measured using items adapted from the Stanford Expectancies of Treatment Scale (Younger et al. 2012). Finally, questions related to consumers' behavioural intentions to receive PAT included the following two items: ‘would you like to receive PAT?’ and ‘would you prefer PAT over conventional talking therapies’, which were measured using a continuous scale ranging from 1 (‘1 = not at all’ to ‘10 = definitely’). The questionnaire was co‐designed through a process of iteratively and collaboratively determining the construct validity of each item with researchers, lived experienced researchers and a consumer advisory group. A 5‐item Likert‐type responses were used throughout.

### 2.3 Participant Recruitment

Participants were recruited to the study through advertisements displayed on social media and distributed in paper form to clinics, via group emails and ‘snow balling’. Advertisements contained information about how to access the online survey and indicated that researchers were interested in capturing knowledge, beliefs and motivations behind interest (or disinterest) towards psychedelic‐assisted therapy from a mental health consumer perspective. Those who chose to access this survey were directed to the participant information sheet and consent page. Recruitment was limited to Australia since it coincides with the TGA downscheduling decision. Once consent was obtained, participants were automatically directed to the survey. By way of screening, preliminary questions included eligibility criteria (which included being aged between 18 and 65 years and identify as a mental health consumer). If these criteria were met, the participants were directed to the remaining questions. All data were captured on REDCap secure web platform where the survey was hosted (Harris et al. 2009).

### 2.4 Data Analysis

Questionnaire data were exported from REDCap software version 13.4.10 and analysed using SPSS version 24. Unadjusted linear regression models were used to evaluate associations between demographic variables and mean acceptability scores. Adjusted regression models were used to further evaluate the association between personal experience with psychedelics and acceptability scores and behavioural intentions to receive PAT. For increased clarity in reporting, the findings in acceptability of PAT, 5‐item Likert scales were collapsed into 3‐level categorical variables (agree, neutral and disagree). The 5‐item Likert scale survey items that were negatively worded were reverse coded before scoring. The Benjamini–Hochberg method was used to control the type I error‐rate associated with multiple testing (Benjamini and Hochberg 1995). Collinearity between variables in the models was assessed using variance inflation factors (VIFs), with a VIF > 10 indicating high collinearity. No collinearity was found between the variables.

## 3 Results

### 3.1 Sample Characteristics

Of the 336 people who accessed the survey, 254 met the inclusion criteria and completed the survey. Baseline characteristics are displayed in Table 1. The overall mean age was 42.5 years, and 79.1% were female. Almost all participants (94.9%) had previously accessed mental health treatment, with a moderate perception of treatment effectiveness (55.4%) and relative comfort with the treatment provider (67.8%). The most common primary mental health diagnoses were depression (33%) and post‐traumatic stress disorder (22%), followed by generalised anxiety disorder (12%). In terms of treatment settings, participants had most often seen psychologists (33%), or had been to a hospital (18%) or community mental health service (14%). A considerable portion of the sample had a comorbid substance use problem (40.6%, with alcohol use being the most common substance use problem) and approximately one third of participants had used psychedelics previously. LSD (22%), psilocybin (18.5%) and MDMA (16.5%) were the most common psychedelics used in the past, and ‘connection’ (54%) and ‘mood’ (40%) were the most common reasons given for participants' motivation to use psychedelics, whilst a smaller proportion of participants specified ‘performance’ as a motive (24%). Although 75% of the sample expressed a desire to access PAT, and 55% stated that they would prefer it over conventional therapy, only 1.7% of the sample had previously experienced PAT.

**TABLE 1:** Sample characteristics.

Characteristics	(*n* = 254)
Mean age, years (SD)	42.4 (12.8)
Gender, % F	79.1
Previous mental health treatment %	94.9
Mean treatment effectiveness %	55.4
Mean treatment provider comfort %	67.8
Substance use problem %	40.6
Previous psychedelic use %	31.5
Previous PAT %	1.7
Would like to receive PAT %	75
Would prefer PAT over conventional therapy %	55

### 3.2 Describing Attitudes Towards PATs

#### 3.2.1 Acceptability of PATs

**FIGURE 1:**
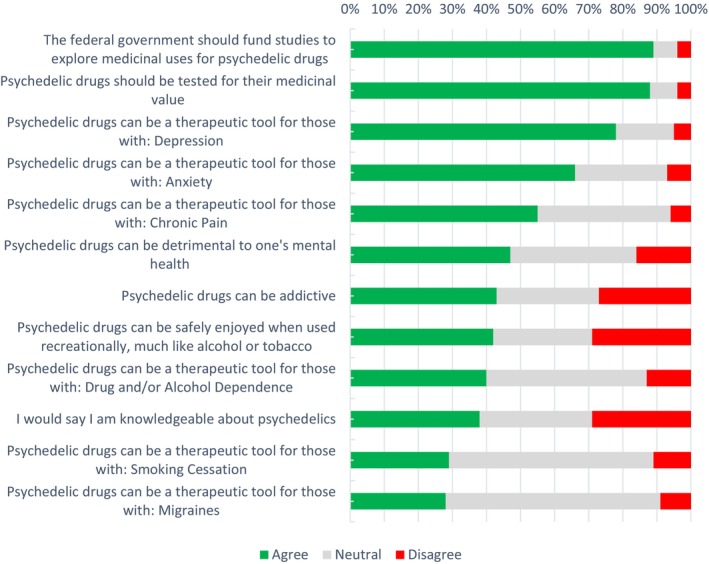
Knowledge and attitudes about the effectiveness of PATs.

**FIGURE 2:**
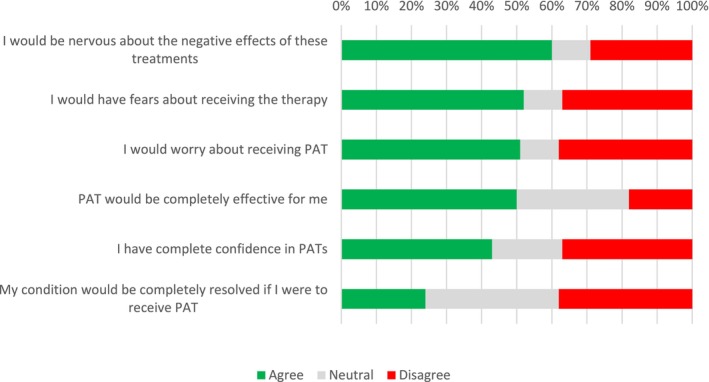
Expectations about the efficacy of PAT.

Survey items related to acceptability of PAT included knowledge and attitudes about the effectiveness of PAT (Figure 1), social norms and expectations about the efficacy of PAT (Figure 2). In each figure, survey items are ranked in order of agreement, from highest to lowest.

Participants displayed the strongest agreement (89%) with the statement ‘The federal government should fund studies to explore medicinal uses for psychedelic drugs’, closely followed by (88%) ‘Psychedelic drugs should be tested for their medicinal value’. Participants also agreed with the therapeutic potential of the use of psychedelics for depression (78%), anxiety (66%) and chronic pain (55%). The lowest agreement was with the therapeutic potential of the use of psychedelics with smoking (29%) and migraines (28%).

The most highly rated social influence was ‘My peers support the use of psychedelics as a therapeutic tool’ (53%), closely followed by *‘People I respect endorse PAT’* (52%), whilst the statement ‘My peers support the use of recreational psychedelics’ was endorsed by a third of the sample (33%).

With regard to expectations about the efficacy of PATs, the three highest rated items had to do with feeling concerned (60%), fearful (52%) and worried (51%) about receiving PAT, although half of the sample believed that PAT would be completely effective for them.

#### 3.2.2 Behavioural Intentions to Access PAT

Twenty nine per cent reported that they would *definitely* like to receive PAT, and approximately one fifth of the sample reported that they would *definitely* like to receive PAT over conventional therapy. Seventy five per cent expressed some desire to receive PAT.

### 3.3 Associations Between Socio‐Demographic Characteristics, Acceptability of and Behavioural Intentions to Try PATs

Univariate consumer characteristics associated with acceptability scores and behavioural intention scores are displayed in Table 2. Younger age was significantly associated with social norms that favour PATs (*d* = −0.47) and males were significantly more likely to expect PATs to be efficacious than females or gender diverse people (*d* = 0.58), whilst females were significantly more likely to doubt the expected efficacy of PATs than males or gender diverse people (*d* = −0.50). Similarly, males were significantly more likely than females or gender diverse people to prefer to receive PAT over conventional talking therapy (*d* = 0.63).

**TABLE 2:** Univariate consumer characteristics associated with acceptability and behavioural intentions.

Variable	Acceptability									Behavioural intentions					
	Attitudes: effectiveness			Expectancy: efficacy			Social norms			Like to receive			Prefer PAT		
	*d*	CI	*p*	*d*	CI	*p*	*d*	CI	*p*	*d*	CI	*p*	*d*	CI	*p*
Age	−0.20	−0.09, 0.01	0.13	0.14	−0.71, 0.10	0.75	−0.47	−0.06, −0.01	0.004	−0.16	−0.22, 0.28	0.82	0.39	−0.001, 0.05	0.06
Gender															
Female	−0.20	−2.99, 0.38	0.13	−0.50	−6.74, −1.47	0.002	−0.29	−1.28, 0.24	0.18	−0.36	−0.12, 0.38	0.32	−0.56	−2.01, −0.44	0.002
Male	0.29	−0.46, 3.36	0.14	0.58	2.61, 8.56	< 0.001	0.24	−0.40, 1.33	0.29	0.47	−0.07, 0.43	0.16	0.63	0.73, 2.49	< 0.001
Gender diverse	0.14	−2.44, 3.54	0.72	−0.14	−5.22, 4.03	0.80	0.20	−0.83, −1.80	0.47	−0.20	−0.20, 0.30	0.70	−0.12	−1.43, 1.31	0.93
Mental health history															
Treatment Effectiveness	−0.31	−0.06, −0.01	0.02	−0.54	−0.12, −0.03	0.001	−0.18	−0.02, 0.01	0.50	−1.32	0.26, 0.78	< 0.001	−0.63	−0.04, −0.01	< 0.001
Treatment Provider Comfort	−0.22	−0.05, 0.01	0.10	−0.32	−0.09, 0.01	0.08	−0.30	−0.02, 0.002	0.11	−0.88	0.10, 0.61	0.007	−0.54	−0.04, −0.01	0.004
Previous psychedelic use															
Any use	0.72	2.01, 4.85	< 0.001	0.80	3.80, 8.24	< 0.001	0.63	0.70, 1.99	< 0.001	1.14	0.19, 0.70	0.001	0.61	0.51, 1.87	< 0.001
Positive Experience of Psychedelics	1.32	0.07, 0.15	< 0.001	1.32	0.15, 0.29	< 0.001	0.82	0.01, 0.05	0.003	1.32	0.56, 1.50	< 0.001	0.70	0.003, 0.05	0.03

Abbreviations: CI, 95% confidence intervals; *d*, effect size; *p*, *p*‐value.

In terms of mental health history, participants who evaluated conventional psychosocial treatment experiences more favourably had significantly less confidence in the effectiveness (*d* = −0.31) and expected efficacy (*d* = −0.54) of PATs, were significantly less likely to choose PATs over conventional therapy (*d* = −0.63) and significantly less likely to want to receive PAT (*d* = −1.32). Participants who reported increased comfort with conventional therapy treatment providers were also significantly less likely to choose PATs over conventional therapy (*d* = −0.54) and significantly less likely to want to receive PAT (*d* = −0.88).

By contrast, participants with previous experience using psychedelics were significantly more likely to report positive attitudes towards the effectiveness (*d* = 0.72) and the expected efficacy of PATs (*d* = 0.80), had social norms that significantly favoured PATs (*d* = 0.63), expressed a significant preference for receiving PATs over conventional therapy (*d* = 0.61) and a significant desire to receive PAT (*d* = 1.14). This was particularly evident for those who had reported more positive experiences of psychedelic use.

### 3.4 Associations Between Acceptability and Behavioural Intentions

Multivariate models of the acceptability of PAT and behavioural intentions to try PAT are presented in Table 3. After controlling for age, gender, previous psychedelic use and effectiveness of conventional therapy, higher efficacy scores, more supportive social norms and the desire to receive PAT were significantly associated with stronger agreement about the *effectiveness* of PAT (Acceptability Model 1; *d =* 0.39–1.12). After controlling for age, social norms, previous psychedelic use and effectiveness of conventional therapy, being male, higher effectiveness scores and the desire to receive PAT were significantly associated with stronger agreement about the *efficacy* of PATs (Acceptability Model 2; *d =* 0.39–1.09). After controlling for gender, previous psychedelic use, effectiveness of conventional therapy and efficacy scores, younger age (*d* = −0.43), stronger agreement about the effectiveness of PATs (*d =* 0.54) and the desire to receive PAT (*d =* 0.54) were significantly associated with more supportive *social norms* (Acceptability Model 3). After controlling for age, gender and previous psychedelic use, reduced effectiveness of conventional therapy (*d = −*0.31) and stronger agreement with acceptability scores related to effectiveness, efficacy and social norms were significantly associated with the desire to receive PAT (Model 4; *d =* 0.37–1.32).

**TABLE 3:** Multivariate linear regression models for acceptability of PATs and behavioural intentions to receive PAT.

	Acceptability												Model 4: Like to receive PAT			
	Model 1: Effectiveness				Model 2: Efficacy				Model 3: Social norms							
Variable	*d*	CI	*BH‐p*	VIF	*d*	CI	*BH‐p*	VIF	*d*	CI	*BH‐p*	VIF	*d*	CI	*BH‐p*	VIF
Age	−0.22	−0.07, 0.02	0.27	1.14	0.24	−0.01, 0.11	0.13	1.13	−0.43	−2.74, 0.01	0.03	1.11	0.16	−0.01, 0.03	0.67	1.14
Gender																
Female	0.24	0.08, 0.55	0.27	1.46	—	—	—	—	—	—	—	—	—	—	—	—
Male	—	—	—	—	0.39	1.54, 5.61	0.003	1.05	0.12	−0.79, 0.85	0.98	1.10	−0.24	−1.25, 0.20	0.25	1.09
Gender diverse	0.24	−0.87, 4.11	0.27	1.47	−0.12	−3.39, 2.55	0.78	1.06	−0.10	−1.18, 1.15	0.98	1.06	−0.16	−1.36, 0.71	0.67	1.06
Personal experience																
Psychedelic use—Yes	0.14	−0.13, 2.14	0.16	1.15	0.26	−0.18, 3.01	0.13	1.15	0.26	−0.19, 1.07	0.23	1.15	−0.12	−0.60, 0.52	0.89	1.16
Conventional therapy—Effectiveness	0.14	−0.02, 0.03	0.73	1.12	−0.24	−0.06, 0.01	0.13	1.11	0.24	−0.01, 0.02	0.14	1.12	−0.31	−0.02, −0.001	0.05	1.10
Acceptability of PAT																
Effectiveness	—	—	—	—	0.85	0.38, 0.72	0.003	1.83	0.54	0.02, 0.17	0.03	2.09	0.54	0.05, 0.17	0.004	2.01
Efficacy	1.12	0.19, 0.36	0.004	2.38	—	—	—	—	0.41	−0.01, 0.10	0.18	2.77	1.32	0.12, 0.20	0.004	2.19
Social Norms	0.39	0.08, 0.55	0.02	1.39	0.26	−0.06, 0.60	0.13	1.42	—	—	—	—	0.37	−0.03, 0.26	0.03	1.40
Like to receive PAT	0.58	0.19, 0.71	0.004	2.24	1.09	1.00, 1.66	0.003	1.85	0.54	0.04, 0.33	0.03	2.29	—	—	—	—

Abbreviations: *BH‐P*, Benjamini–Hochberg adjusted *p*‐value; CI, 95% confidence intervals; *d*, effect size.

## 4 Discussion

The current momentum towards the translation of PATs into widespread use must be tempered by research into its acceptability as well as efficacy and feasibility. The present findings suggest that PATs are generally acceptable to mental health consumers and that intentions to try PAT are both guided by these beliefs and related to less satisfying experiences of conventional therapy (regardless of previous psychedelic use). The key aim of this study was to enhance current knowledge about the implementability of PATs amongst mental health consumers by: (1) describing its acceptability (attitudes, social influences and expectancy), (2) identifying demographic characteristics and personal experiences (mental health, substance use, psychedelic use and conventional mental health treatment) that might influence perceptions of acceptability and (3) exploring potential predictors of PAT uptake through the utilisation of the TPB.

Mental health consumers in this study reported moderate satisfaction with conventional therapy and three quarters of the sample expressed a desire to access PAT if it became available. Just over half the sample reported that they would prefer PAT over conventional talking therapy, which is similar to the findings of Corrigan et al. (2021) that 55% of their sample would be willing to accept psilocybin therapy as a replacement to current medications, if recommended by a doctor. Attitudes about the importance of testing psychedelics for their medicinal value were also similar to Corrigan et al. (2021), but agreement about the necessity of government funding for research exploring the medicinal uses of psychedelics was much stronger in this population (89% vs. 52%). Participants in this study displayed stronger agreement with the effectiveness of PATs than the Corrigan et al. (2021) sample and the Nadeem et al. (2024) sample, and some evidence of social norms favouring the use of PATs. Many consumers indicated that they would feel nervous about possible adverse effects of PATs, which is a common theme across similar studies (e.g., Corrigan et al. 2021; Jilka et al. 2019; Jilka et al. 2021; Harding et al. 2021; Nadeem et al. 2024), whilst half of the sample believed that PAT would be *completely effective* for them. One third of the sample had used psychedelics previously.

Although univariate analyses yielded findings consistent with Corrigan et al. (2021) regarding the relationships between the endorsement of PATs and previous psychedelic use, male gender and younger age and Nadeem et al. (2024) regarding the relationship between previous psychedelic use and endorsement of PATs, multivariate analyses revealed that gender (79% female) and age were associated with some aspects of acceptability but that none of these factors were associated with the desire to receive PAT. On the contrary, higher acceptability scores and more negative experiences of conventional therapy were associated with PAT desirability. When interpreted in light of the Theory of Planned Behaviour, these findings may be used to predict PAT adoption since more favourable attitudes towards effectiveness, beliefs about efficacy and social norms were found to influence intentions to try PAT. It appears that mental health consumers who are looking for an alternative to conventional therapy might be more willing to try PAT, provided they feel confident in its efficacy and effectiveness, and provided they have the support of their peers.

From an implementation perspective, it is also worth considering the large group of consumers who expressed fears or concerns about the efficacy of PATs (nervous = 60%, fearful = 52% and worried = 51%) and the stigma that may be present where social norms do not support PAT use (11%–13%). Stigma has been found to influence consumers' willingness to disclose information about personal psychedelic use to providers (Boehnke et al. 2023) as well as specific concerns related to the therapeutic use of psychedelics. For instance, research on perspectives of psychedelic treatment in eating disorders found that consumers were worried about becoming addicted to the therapeutic drug (61%), long‐term psychological effects (61%), negative psychedelic experiences (60%), long‐term physical health effects (55%) and other side effects (54%), with just under half of the consumers concerned about stigma (46%). Interestingly, knowledge about licensing of some psychedelics for mental health conditions ameliorated concerns in over half of consumers with eating disorders (54.3%) (Harding et al. 2021). Studies of consumer views of the clinical use of ketamine have also highlighted concerns about side effects and have signalled the importance of providing information about the effects of ketamine, any side effects, long‐term effectiveness and costs (Jilka et al. 2019, 2021). Stigma was identified by a considerable number of consumers in this research, particularly due to the bad reputation of ketamine as a ‘horse tranquiliser’ and hallucinogenic drug taken in night clubs (Jilka et al. 2019). Stigma surrounding the therapeutic use of psychedelics has also been identified amongst consumers with a diagnosis of Fibromyalgia, and may be a stronger barrier for those who are ‘psychedelic naïve’ (Glynos et al. 2022).

### 4.1 Strengths and Limitations

This study has introduced an increased rigour to the study of attitudes and perceptions of PATs amongst mental health consumers by incorporating measures of acceptability and behavioural intentions and drawing on the TPB. The study was administered during a period of time when drug scheduling changes were underway in Australia and provides timely data to assist with the successful implementation of PATs following the TGA downscheduling decision. Limitations include the potential for self‐selection bias common to web‐based surveys and the cross‐sectional study design, which limits the inferences that can be made about the findings and can be more pronounced when advertisements mention that the study is about psychedelics. The inclusion of Ketamine is another limitation, given that it has been used therapeutically for many years and may attract more favourable attitudes. There was also an overrepresentation of females in the sample (a pattern which is often observed when online recruitment methods are used; Choi et al. 2017) and the data relied on self‐reported history of drug use.

## 5 Conclusion

Australia appears set to incorporate PATs as treatment options for mental health and substance use disorders. Successful translation of PATs will require high quality information about their efficacy, feasibility and acceptability at various levels of implementation (policy, organisational, clinician and consumer). This study has utilised the TPB to provide insights into the implementability of PATs amongst Australian mental health consumers through an evaluation of its acceptability and desirability. Consumers in this study were generally open to PATs, despite some concerns, and had strong beliefs about its effectiveness. Acceptability appears to play an important role in consumer willingness to try PATs, regardless of previous psychedelic use, although some concerns need to be addressed. PAT treatment appears unacceptable to a number of consumers, with up to 60% of the sample expressing concerns about its expected efficacy. A person‐centred approach might assist with communicating information about PATs with consumers, whilst minimising the effects of stigma. As PATs become more readily available to mental health consumers, research into other aspects of acceptability (e.g., cost, access, burden, intervention coherence and opportunity costs) will be useful.

## 6 Relevance for Clinical Practice

Government support has been granted for certain PATs in Australia, and clinicians will play a crucial role in the implementation of PATs. As evidence accumulates on the efficacy, safety and sustainability of PATs, training and accreditation of clinicians will also be necessary, and knowledge about the mental health consumer attitudes will help inform best practice. Findings from this study suggest that PATs might be desirable amongst those seeking an alternative to conventional therapy and that a person‐centred approach is recommended.

## Author Contributions

E.L. and K.M. conceptualised, lead and designed the study. E.L., E.T., J.W., Z.B. and K.M. co‐designed the measures. E.L. and E.T. contributed to study coordination, recruitment, data collection and data maintenance. E.L. and K.M. contributed to data analysis and interpretation, and writing of the manuscript. Additional oversight and expert contributions were made by P.H. and A.R.M. All authors have approved the final manuscript.

## Ethics Statement

Ethics approval was obtained from the Sydney Local Health District Royal Prince Alfred Hospital (SLHD‐RPA) ethics committee. Protocol No: X22‐0243 and 2022/ETH01223. Completing and submitting the survey implied consent.

## Conflicts of Interest

The authors declare no conflicts of interest.

## Data Availability

The data that support the findings of this study are available on request from the corresponding author. The data are not publicly available due to privacy or ethical restrictions.
